# Local anaesthesia vs. general anaesthesia for percutaneous microwave ablation in hepatocellular carcinoma: efficacy, safety, and cost analysis

**DOI:** 10.3389/fonc.2023.1186133

**Published:** 2023-09-12

**Authors:** Jing-Ying Zhan, Dan Zhao, Zhen-Lei Tang, Hao-Qun Leng

**Affiliations:** ^1^ Department of Radiology, The Second Xiangya Hospital of Central South University, Changsha, China; ^2^ Department of Anesthesiology, Peking University Third Hospital, Beijing, China

**Keywords:** hepatocellular carcinoma, microwave ablation, anaesthesia modality, efficacy, cost and safety analysis, propensity score matching

## Abstract

**Purpose:**

To compare the efficacy, safety, and cost of local anaesthesia and general anaesthesia modalities for percutaneous microwave ablation as a curative treatment for hepatocellular carcinoma patients.

**Methods:**

This comparative, retrospective study analysed 175 patients who were treated for hepatocellular carcinoma (HCC) from July 2015 to September 2020. Conventional transcatheter arterial chemoembolization (cTACE) combined with sequential percutaneous microwave ablation (MWA) was performed on every lesion in every patient. Patients were divided into two cohorts according to the anaesthesia modality applied during MWA. To investigate the differences in efficacy between the two groups, overall survival (OS) and local recurrence-free survival (LRFS) were estimated by the Kaplan−Meier method and compared by the log-rank test. Cost and safety between the two groups were also compared accordingly.

**Results:**

There were 105 patients with 128 HCC lesions in the local anaesthesia (LA) group and 70 patients with 107 lesions in the general anaesthesia (GA) group. There were no significant differences in OS (P = 0.798) or LRFS (P = 0.406) between the two groups. Fifty-two pairs of patients were matched with 77 lesions in the GA group and 67 lesions in the LA group after PSM. There was no significant difference in OS (P = 0.522) or LRFS (P = 0.410) between the two groups. Compared to the LA group, the GA group had longer operations, consumed more medical resources, had a heavier financial burden, and experienced more anaesthesia adverse events. There was no significant difference in the incidence of post-ablation pain (p=0.487), fever (P=0.678), nausea or vomiting (P=0.808), mild liver dysfunction (P=0.753), haemolytic uraemic syndrome (P=0.595), pleural effusion (P=0.622), liver abscess (0.544), asymptomatic perihepatic fluid (0.703) or subcapsular liver hemorrhage (P=0.666) between the two groups.

**Conclusion:**

Due to the higher cost and adverse events of general anaesthesia, local anaesthesia may be more suitable for ablation procedures for HCC patients within the Milan criteria.

## Background

Liver cancer remains a global health challenge worldwide while hepatocellular carcinoma (HCC) accounts for approximately 90% of all liver cancer cases (1–3). Percutaneous ablation is recommended as a curative therapy for patients with early-stage HCC who are not candidates for surgical resection and liver transplantation (4). Moreover, the scope of ablation of HCC can be extended by combining adjunctive methods such as transcatheter arterial chemoembolization (TACE), and the treatment efficacy has been verified in several studies (5–7). Therefore, percutaneous ablation is widely applied as curative therapy for early HCC.

Pain management during and after the ablation procedure remains a major challenge in clinical practice. Intraoperative and postoperative pain, anxiety, and intraoperative respiratory movement may affect the treatment efficacy and safety of ablation. Currently, local anaesthesia (LA) combined with intravenous sedation or general anaesthesia (GA) is applied most frequently during the ablation procedure, but the most appropriate anaesthesia method is still a highly debated topic worldwide (8). A few studies have compared different anaesthesia modalities for ablation of liver cancer. However, no clinical study has compared different anaesthesia methods in MWA procedure for HCC patients who received cTACE firstly as sequential therapy.

Therefore, the purpose of the present study was to investigate the treatment efficacy, safety, and cost of general anaesthesia and local anaesthesia combined with sedation during the ablation procedure for HCC patients who received combination therapy (TACE plus ablation) for curative treatment purposes.

## Materials and methods

### Study design and population

This retrospective study was approved by the institutional review board of our hospitals and was performed according to the Declaration of Helsinki. Due to the retrospective nature of the present study, the requirement for written informed consent was waived by the institutional review boards.

**Figure 1:**
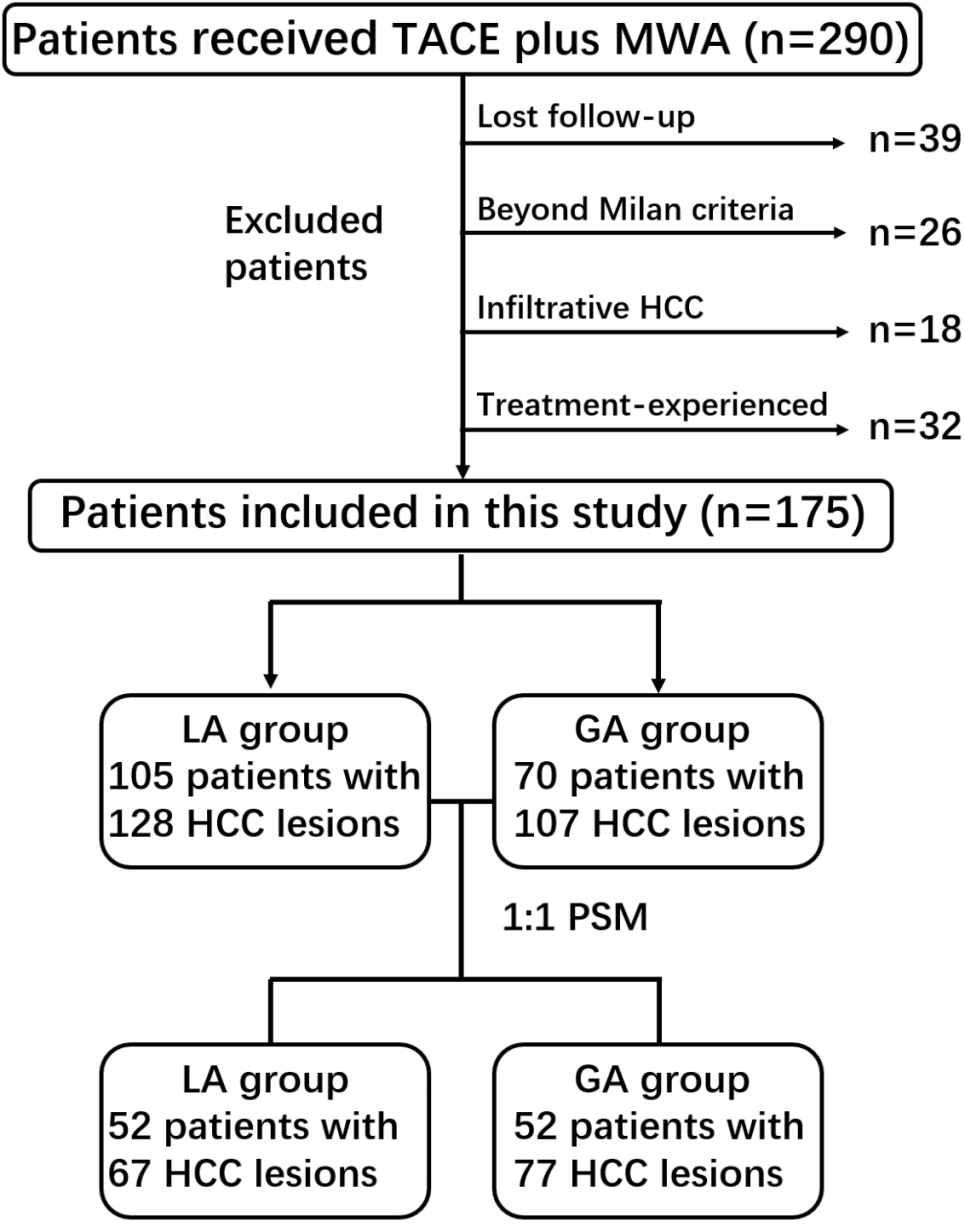
Flowchart of the study population.

From July 2015 to September 2020, a total of 290 consecutive patients with unresectable HCC who received cTACE plus MWA were included. The choice of cTACE plus MWA was made on a case-by-case basis by the multidisciplinary treatment board (consisting of interventional radiologists, medical oncologists and liver surgeons) and after in-depth discussion with the patient himself/herself. Patients who meet the following criteria were included in this study: (a) diagnosed with HCC by Li-RADS 5 category or biopsy; (b) no macrovascular invasion or distant metastasis; (c) preserved liver function (Child−Pugh A or B); and (d) an Eastern Cooperative Oncology Group performance status of 0. The exclusion criteria included (a) lost to follow-up (n = 39); (b) beyond the Milan criteria (single lesion within 5 cm or no more than three nodules with the largest lesion within 3 cm) (n = 26); (c) infiltrative HCC (n = 18); and (d) received other treatment for HCC lesions before cTACE plus MWA (n = 32). Finally, 175 patients with 235 HCC lesions were included in the present study. The flowchart of the study population is shown in **Figure 1**.

### cTACE procedure

cTACE was performed by four board-certified senior interventional radiologists. The femoral artery was routinely catheterized and digital subtraction angiography (DSA) of the superior mesenteric- and celiac arteries was performed using a 5-Fr RH catheter (Terumo Co. Ltd, Tokyo, Japan). After hepatic arteriography, a coaxial microcatheter was placed as superselectively as possible in the tumour feeders to slowly inject the emulsion of iodized oil (Lipiodol, Guerbet Group, France) and epirubicin/doxorubicin. The Lipiodol-epirubicin/doxorubicin emulsion was created by a mixing up to 15 mL of Lipiodol and distilled water, dissolving 50 mg to 120 mg of epirubicin or 100 mg of doxorubicin at a ratio of 3:1 or 2:1, respectively. The gelfoam slurry was injected through the microcatheter to embolize the proximal tumour feeders. All procedures were technically successful according to the Society of Interventional Radiology (SIR) guidelines (9).

### Anaesthesia modality

The preanaesthesia evaluation was performed according to the American Society of Anaesthesiologists (ASA) physical status classification system for patients in the GA group (10). All patients were ASA physical status I or II. Sedation, laryngeal mask insertion, monitoring of haemodynamics, and electrocardiogram were performed during general anaesthesia. The GA group was given propofol (1mg/Kg), midazolam (0.02-0.03mg/kg), and fentanyl (1 to 2μg/kg) via the veins to induce anaesthesia, fitted with a laryngeal mask and placed under mechanical ventilation. Propofol (5 to 8 mg/kg/h) was used intravenously to maintain the depth of general anaesthesia. All the drug doses used during the ablation procedure in the GA group were commissioned by a certified anaesthesiologist. The patients in the GA group recovered from general anaesthesia in the postanaesthesia care unit after the MWA procedure was finished.

In the LA plus intravenous sedation group (Hereinafter abbreviated as the LA group), approximately 10 mL of 2% lidocaine was injected subcutaneously at the puncture point. A unit of midazolam and fentanyl, as the starting dose of sedatives, was intravenously injected to control intraoperative pain and anxiety. During MWA, the sedative dose would increase according to the operation time and the patients’ pain level. Haemodynamic and electrocardiogram monitoring was performed, and the patient breathed spontaneously while awake during the operation.

### Percutaneous microwave ablation procedure

All tumours were percutaneously ablated within 3 days of embolization of the tumour vessels with iodized oil and gelatine sponges, and cTACE plus the following MWA were performed during a single hospitalization. Percutaneous microwave ablation was performed under microwave ablation (MWA) systems (KY-2000, Kangyou Medical Instrument Co. Ltd., China) by two board-certified senior interventional radiologists (one with more than 15 years of experience in percutaneous ablation and another with 9 years). Before the insertion of the ablation needle, unenhanced computed tomography (CT) was carried out and previous imaging data were reviewed, and then the antenna was percutaneously inserted into the lesion under CT guidance. The overlapping ablation technique was performed for tumour lesions larger than 3.0 cm. The MWA was set from 60 W to 140 W, and the ablation time was 3 min to 15 min. If necessary, artificial ascites were created through a fine hollow needle, especially for lesions in subcapsular locations. The respiratory motion of the patients in the GA group was regulated with the help of an anesthesiologist during the ablation procedures. The MWA procedure time was defined as the time from the patient’s arrival to their exit from the operating room.

**Figure 2:**
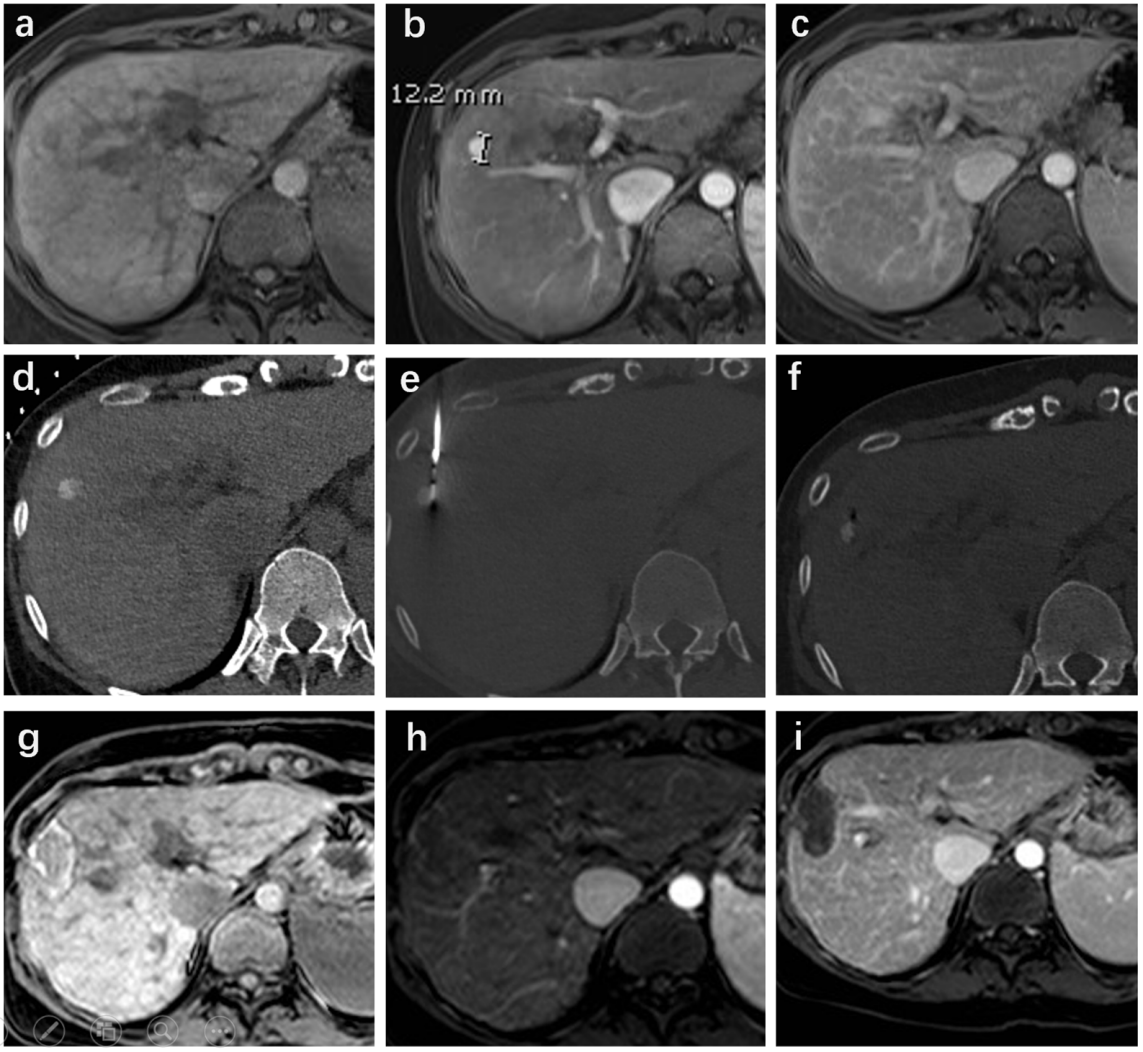
A 63-year-old female with hepatitis B was diagnosed with Li-Rads 5 hepatocellular carcinoma (HCC) nodular in segment VIII. The pre-ablation magnetic resonance imaging (MRI) showed the lesion located in the right lobe with non-rim enhancement in the arterial phase (maximum diameter of 12 mm) and non-peripheral washout with an enhancing capsule in the delayed phase **(A-C)**. The lineament of the HCC lesion with iodized oil retention was well depicted in the CT scan **(D)**. CT-guided microwave ablation (MWA) was performed under local anaesthesia plus sedation **(E, F)**. Follow-up MRI was performed four weeks after ablation and showed a complete response **(G-I)**.

Contrast-enhanced multiphase CT (including arterial, portal, and delayed phases) was performed immediately after ablation for patients with indistinct ablation margins, and immediate complications and technical success were assessed. The technical success of MWA was defined as complete ablation of the tumour with a safety margin of at least 0.5 cm on CT images. For residual viable tumours, repeated ablation procedure was performed until technical success was achieved. **Figure 2** presents a typical case of a HCC patient who received MWA after cTACE.

### Data collection

The demographic, laboratory, and radiological data before cTACE plus MWA were collected for each patient, including age (≤ 59 years/>59 years), gender (male/female), Child−Pugh class (A/B), etiology of hepatitis (none/hepatitis B/hepatitis C/alcohol/others), serum alpha-fetoprotein (AFP) level (≤ 400 ng/mL/>400 ng/mL), cirrhosis (presence/absence), number of tumours (single/multiple), tumour size (≤ 3 cm/>3 cm), perivascular (yes/no) and subcapsular (yes/no). Patients were stratified based on the median age (59 years) in the present cohort. Perivascular HCC lesions were defined as lesions adjacent to larger vessels (> 3 mm) while subcapsular lesions were located within 1 mm of the liver capsule (11, 12). MWA procedure-related data, including duration of the MWA procedure, number of healthcare providers participating, and adverse events (AEs) or complications, were also recorded. The duration of the MWA procedure was defined as the time between the patient entering and leaving the operation room. The intra- (only in the LA group) and post-ablation pain (within 24 h after the MWA procedure) was recorded, and classified as mild pain (5–44 mm), moderate pain (45–74 mm), or severe pain (75–100 mm) according to the VAS criteria (13). We also recorded the duration of the hospital stay and the hospitalization costs for every patient.

### Definitions and follow up

Local tumour recurrence (LTR) was defined as the appearance of viable tumour foci at the edge of the ablation zones after complete response (CR) in at least one contrast-enhanced follow-up image. Local recurrence free survival (LRFS) was defined as the time from ablation to LTR of the target lesion or the last imaging follow-up (classified as censored data). Overall survival (OS) was the time between the first ablation and death from any cause or the last follow-up (classified as censored data) (Oct 03, 2021). Ablation-related complications were jointly evaluated according to the National Cancer Institute Common Terminology Criteria for Adverse Events (CTCAE Version 5.0) (14) and the Society of Interventional Radiology (SIR) classification system (15).

Patients were followed up at 1, 3, 6, 9, and 12 months after the cTACE plus MWA procedure in the first year and then every 6 months thereafter. At each follow-up visit, contrast-enhanced magnetic resonance (MR) imaging of the liver was performed to evaluate LTR. The choice of treatment modality for recurrent HCC was dependent on the site of the tumour, liver function, and the general condition of the patient. The primary endpoint of our study was LRFS for lesions, and the secondary endpoint was OS for patients. The ablation evaluation standards were based on the modified response evaluation criteria in solid tumours (mRECIST) guidelines (16).

### Statistical analysis

Categorical variables were compared using the χ2 test or Fisher’s exact test, as appropriate. Continuous variables were compared using the Mann−Whitney U test or t test accordingly. To diminish the potential confounding and selection bias of the two groups, propensity score matching (PSM) methods were applied. PSM is a statistical matching technique that attempts to reduce the bias due to confounding variables that could be found in an estimate of the treatment effect (17). All factors that may affect the outcome of the ablation procedure were included for propensity score matching. The GA and LA groups were matched by using a one-to-one PSM method with a caliper width of 0.2. LRFS for lesions and OS for patients between the two groups were estimated by the Kaplan−Meier method and compared by the log-rank test. Stratification analyses were performed, to compare LRFS, in the perivascular (yes/no) and subcapsular (yes/no) subgroups after PSM. The differences in safety and cost between the GA and LA groups were appropriately analysed before and after PSM. A two-sided P value less than 0.05 indicated statistical significance. Statistical software (SPSS version 24, International Business Machines Corporation, USA) or R software (version 4.0.2, http://www.R-project.org) was used for statistical analysis.

## Results

### Baseline patient characteristics

A total of 175 treatment-naïve HCC patients with 235 lesions were enrolled in our study, including 142 males and 33 females, with a mean age of 59.2 ± 11.3 years, ranging from 27 to 83 years. There were 105 patients with hepatitis B, 20 patients with hepatitis C, 15 patients with alcoholic hepatitis, 18 patients with other aetiologies of hepatitis (steatohepatitis (n=13) and cryptogenic (n=5)), and 17 patients without basic hepatitis. Patients were diagnosed with HCC based on pathological assessment (n=21) or the 2018 version of the LI-RADS criteria (n=154). There were 148 patients with Child−Pugh A and 27 patients with Child−Pugh B. In the entire study population, there were 70 patients with 107 HCC lesions in the GA group, and the LA group included 105 patients with 128 lesions. After one-to-one PSM, 52 pairs of patients were matched, with 77 lesions in the GA group and 67 lesions in the LA group. The detailed baseline characteristics between the GA and LA groups before and after PSM are illustrated in **Table 1**.

**Table 1:** Baseline characteristics of GA group and LA group before PSM and after PSM.

Characteristics	Before PSM			After PSM		
	GA group (n=70)	LA group (n=105)	P	GA group (n=52)	LA group (n=52)	P
Gender			0.040			0.587
Male	62	80		45	43	
Female	8	25		7	9	
Age			0.267			0.239
≤59	38	48		29	23	
>59	32	57		23	29	
Number of tumors			<0.001			0.292
single	37	86		33	38	
multiple	33	19		19	14	
Tumor size			0.267			0.534
≤3 cm	43	73		33	36	
>3 cm	27	32		19	16	
Perivascular			0.497			0.695
Yes	33	55		26	28	
No	37	50		26	24	
Subcapsular			0.014			0.163
Yes	49	54		34	27	
No	21	51		18	25	
Child−Pugh score			0.698			0.604
A	58	90		42	44	
B	12	15		10	8	
Aetiologies of hepatitis			0.011			0.441
None	3	14		3	4	
HBV	53	52		36	31	
HCV	4	16		3	9	
Alcohol	4	11		4	3	
Others	6	12		6	5	
Cirrhosis			<0.001			0.365
Yes	43	93		37	41	
No	27	12		15	11	
AFP			0.584			1.000
≤400 ng/mL	62	90		45	45	
>400 ng/mL	8	15		7	7	

In patients with multiple lesions, the characteristics of the largest lesion were used for analysis.

GA, General anesthesia; LA, Local anesthesia; PSM, Propensity score matching; AFP, Alpha fetoprotein.

### Comparison of LRFS and OS between GA and LA group before and after PSM

**Figure 3:**
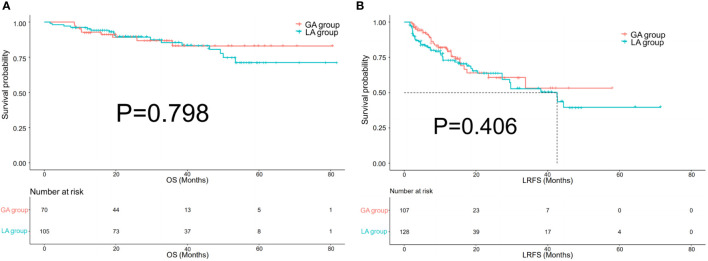
Kaplan−Meier overall survival (OS) curves **(A)** of patients and local recurrence-free survival (LRFS) curves **(B)** of lesions in the GA group and LA group before propensity score matching (PSM).

**Figure 4:**
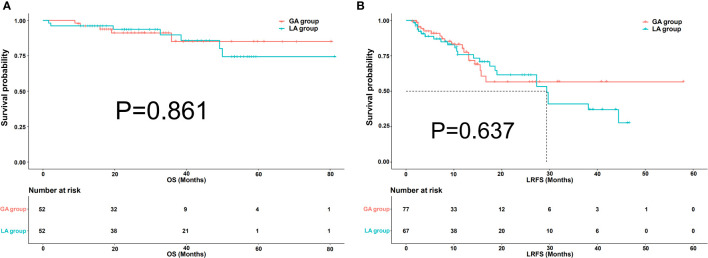
Kaplan−Meier overall survival (OS) curves **(A)** of patients and local recurrence-free survival (LRFS) curves **(B)** of lesions in the GA group and LA group after propensity score matching (PSM).

There were 70 patients with 107 HCC lesions in the GA group and 105 patients with 128 lesions in the LA group before PSM. There was no difference in OS between the two groups, with the same 1-year survival rate (92% vs. 94%, P=0.798). No significant difference in LRFS was observed between the GA and LA groups, with P=0.406. After a one-to-one PSM analysis, there were 52 pairs of matched patients with 77 lesions in the GA group and 67 lesions in the LA group. No significant difference was observed in OS (P=0.861) or LRFS (P=0.637) between the two groups after PSM analysis. **Figures 3**, **4** demonstrate the survival curves of OS and LRFS between the GA and LA groups before and after PSM.

### Subgroup analysis

**Figure 5:**
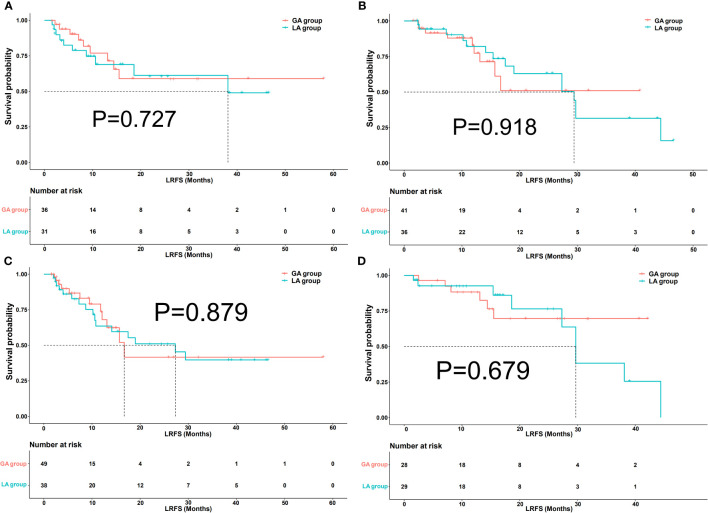
Kaplan−Meier local recurrence-free survival (LRFS) curves of lesions in perivascular **(A)**, non-perivascular **(B)**, subcapsular **(C)**, and non-subcapsular **(D)** locations between the GA group and LA group after propensity score matching (PSM).

After a one-to-one PSM analysis, 77 lesions in the GA group and 67 lesions in the LA group were matched. In the subgroup of perivascular lesions, there were no significant differences in LRFS between the GA and LA groups (P=0.727) (**Figure 5A**). Meanwhile, there was no significant difference in LRFS among the non-perivascular lesions between the GA and LA groups, with P=0.918 (**Figure 5B**). In the subgroup of lesions with and without subcapsular location, both lesions with subcapsular location (P=0.879) (**Figure 5C**) and without subcapsular location (P = 0.679) (**Figure 5D**) demonstrated no differences in LRFS between the GA and LA groups.

### Cost analysis

Costs associated with the MWA procedure time, medical resource consumption, and financial stress undertaken by each patient were recorded accordingly. The MWA procedure time for the GA group (133.8 ± 26.0 mins) was significantly longer than that for the LA group (100.3 ± 18.4 mins) (P = 0.017). Furthermore, more healthcare providers participated in the MWA procedure for the GA group (5.2 ± 0.9) than for the LA group (3.0 ± 0.7) (P = 0.003). The hospitalization costs of every patient were reviewed in the electronic medical records; the cost was ¥45.0 ± 4.3k for patients in the GA group and ¥38.8 ± 1.3k for those in the LA group (p<0.001). For the hospital stays, there was no significant difference between the GA group (5.2 ± 1.0 days) and the LA group (5.0 ± 1.2 days) (p=0.390). The cost analysis between the GA and LA groups showed a similar outcome after PSM. The detailed category and information are illustrated in **Tables 2**, **3**.

**Table 2:** Cost analysis between GA and LA group before PSM.

Category	GA group (n=70)	LA group (n=105)	*p*
**MWA procedure time (min)**	133.8±26.0	100.3±18.4	0.017
**Participating healthcare providers**	5.2±0.9	3.0±0.7	0.003
**Hospitalization costs (¥; K)**	45.0±4.3	38.8±1.3	<0.001
**Hospital stays (day)**	5.2±1.0	5.0±1.2	0.390

GA, General anaesthesia; LA, Local anaesthesia; PSM, Propensity score matching; MWA, Microwave Ablation.

**Table 3:** Cost analysis between GA and LA group after PSM.

Category	GA group (n=52)	LA group (n=52)	*p*
**MWA procedure time (min)**	130.58±25.82	97.89±18.42	<0.001
**Participating healthcare providers**	5.23±0.85	2.92±0.71	<0.001
**Hospitalization costs (¥; K)**	45.34±4.17	38.75±1.28	<0.001
**Hospital stays (day)**	5.19±1.03	5.25±1.25	0.789

GA, General anaesthesia; LA, Local anaesthesia; PSM, Propensity score matching; MWA, Microwave Ablation.

### Intra- and postablation pain, AEs and complication

During the MWA procedure, 78, 24 and 3 patients suffered from mild, moderate, and severe pain respectively in the LA group. After the MWA procedure, there were 41, 21 and 8 patients with mild, moderate, and severe pain respectively in the GA group and 52, 37 and 16 patients in the LA group (p=0.487). Fever (with/without treatment) was the most common AE and no significant differences in any of the AEs were observed between the GA and LA groups. Anaesthesia AEs, including dizziness, urinary retention and respiratory depression, occurred more often in the GA group, with no serious anaesthesia AEs (≥ grades 3) occurring in either group. The incidences of Haemolytic uraemic syndrome (4% vs. 5%), pleural effusion (6% vs. 6%), liver abscess (3% vs. 4%), asymptomatic perihepatic fluid (7% vs. 7%), and subcapsular hepatic hemorrhage (3% vs. 3%) were comparable between both groups. Similar results were observed between the GA and LA groups after PSM. All ablation-related AEs and complications accepted heteropathy accordingly, and no life-threatening complications during treatment occurred. The detailed category and information are summarized in **Tables 4**, **5**.

**Table 4:** Intra- and Post-ablation pain, adverse events and complications analysis between GA and LA group before PSM.

Category	GA group (n=70)		LA group (n=105)		*p*
	Grades	Number (%)	Grades	Number (%)	
**Intra-ablation pain**					**—**
	**-**	**-**	Mild	78 (74)	
	**-**	**-**	Moderate	24 (23)	
	**-**	**-**	Severe	3 (3)	
**Post-ablation pain**					0.487^*^
	Mild	41 (59)	Mild	52 (50)	
	Moderate	21 (30)	Moderate	37 (35)	
	Severe	8 (11)	Severe	16 (15)	
Adverse events of MWA					
** Fever, maximum 38°C, no treatment**	I	7 (10)	I	11 (10)	0.919^*^
** Fever, > 38 °C, treatment**	II	52 (74)	II	75 (71)	0.678^*^
** Nausea or vomiting**	II	13 (19)	II	18 (17)	0.808^*^
** Mild liver dysfunction, requiring conservative treatment**	II	29 (41)	II	41 (39)	0.753^*^
Anesthesia adverse events					
** Dizziness**	I-II	9 (13)	I-II	3 (3)	0.010^§^
** Urinary retention**	I-II	12 (17)	I-II	3 (3)	<0.001^§^
** Respiratory depression**	I	5 (7)	I	1 (1)	0.027^§^
Complications					
** Haemolytic uraemic syndrome**	III	3 (4)	III	5 (5)	0.595^§^
** Pleural effusion**	III	4 (6)	III	6 (6)	0.622^§^
** Liver abscess**	III	2 (3)	III	4 (4)	0.544^§^
** Asymptomatic perihepatic fluid**	IV	5 (7)	IV	7 (7)	0.703^*^
** Subcapsular liver hemorrhage**	IV	2 (3)	IV	3 (3)	0.666^§^

Visual Analog Scale (VAS) for Intra- and Post-ablation pain.

National Cancer Institute Common Terminology Criteria for Adverse Event (CTCAE version 4.03).

Society of Interventional Radiology (SIR) classification system for Complications.

Data are numbers of events. Data in parentheses are percentages.

GA, General anesthesia; LA, Local anesthesia; PSM, Propensity score matching; MWA, Microwave Ablation.

*Pearson χ2 test was used. §Fisher exact test was used.

**Table 5:** Post-ablation pain, adverse events and complications analysis between GA and LA group after PSM.

Category	GA group (n=52)		LA group (n=52)		*p*
	Grades	Number (%)	Grades	Number (%)	
**Post-ablation pain**					0.371^*^
	Mild	29 (56)	Mild	22 (42)	
	Moderate	17 (33)	Moderate	21 (40)	
	Severe	6 (11)	Severe	9 (17)	
Adverse events of MWA					
** Fever, maximum 38°C, no treatment**	I	5 (10)	I	6 (12)	0.750^*^
** Fever, > 38 °C, treatment**	II	38 (73)	II	39 (75)	0.823^*^
** Nausea or vomiting**	II	11 (21)	II	10 (19)	0.807^*^
** Mild liver dysfunction, requiring conservative treatment**	II	21 (40)	II	21 (40)	1.000^*^
Anesthesia adverse events					
** Dizziness**	I-II	9 (18)	I-II	1 (2)	0.008^§^
** Urinary retention**	I-II	10 (20)	I-II	2 (4)	0.014^§^
** Respiratory depression**	I	5 (10)	I	1 (2)	0.092^§^
Complications					
** Haemolytic uraemic syndrome**	III	3 (6)	III	1 (2)	0.308^§^
** Pleural effusion**	III	4 (8)	III	5 (10)	0.727^§^
** Liver abscess**	III	1 (2)	III	3 (6)	0.308^§^
** Asymptomatic perihepatic fluid**	IV	3 (6)	IV	4 (8)	0.696^§^
** Subcapsular liver hemorrhage**	IV	1 (2)	IV	0 (0)	0.315^§^

Visual Analog Scale (VAS) for Post-ablation pain.

National Cancer Institute Common Terminology Criteria for Adverse Event (CTCAE version 4.03).

Society of Interventional Radiology (SIR) classification system for Complications.

Data are numbers of events. Data in parentheses are percentages.

GA, General anesthesia; LA, Local anesthesia; PSM, Propensity score matching; MWA, Microwave Ablation.

*Pearson χ2 test was used. §Fisher exact test was used.

## Discussion

Few studies have focused on comparing different anaesthesia methods for ablation of solid tumours, especially primary or secondary liver cancer and lung cancer (18–22). The study by Lai et al. (19) suggested that treatment of small HCC with RFA under GA is associated with a reduced risk of cancer recurrence. Another study conducted by Wang et al. (20) demonstrated that applying GA in the thermal ablation of HCC patients could significantly improve the survival time of patients compared to LA. Some authors also argued that patients under LA suffered from pain and stress during the ablation procedure, thus leading to an insufficient ablation zone (21). On the other hand, the study by Li et al. argued that different anaesthesia methods have no significant effect on treatment-related complications and LTP in HCC patients treated by MR-guided MWA (22). Taken together, whether the anaesthesia modality affects the therapeutic efficacy of percutaneous ablation in HCC patients is still under debate. To the best of our knowledge, this is the first study comparing different anaesthesia methods in the MWA procedure for HCC patients who received cTACE first.

CT-guided percutaneous thermal ablation is a minimally invasive therapy to treat focal tumours by inducing irreversible cellular injury through the application of thermal energy, and MWA is one of the mainstream ablation methods. As a curative therapy for early-stage HCC, the primary purpose of ablation is to completely eradicate all viable malignant cells within the target HCC lesions (23). The treatment efficacy of ablation for relatively small HCC lesions is comparable to that of radical surgical resection (1, 2, 24). The therapeutic scope of ablation can be extended by combining adjunctive methods such as TACE. TACE combined with sequential MWA, allows a larger ablation area and leads to better efficacy and thus become a favourable treatment modality in clinical practice.

In our study, no significant differences were observed in the LRFS and OS rates between the two different anaesthesia modalities. This outcome is mainly due to the following reasons. First, most patients who accepted local anaesthesia plus sedation during the ablation procedure in our study could control their respiratory movement during needle insertion. To achieve a good therapeutic response to MWA, the precise insertion and placement of the ablation antenna are crucial. For patients who were unable to control their respiratory movement, multiple punctures were performed until the ablation antenna was inserted into the desired position. Second, cTACE performed before MWA served as an adjunctive method, and the lipiodol deposited in the HCC lesions is a conspicuous marker during the MWA procedure. According to the lipiodol label, the ablation antenna could be inserted through the centre of HCC lesions in most cases regardless of the anaesthesia modality. Moreover, TACE before MWA could diminish the blood flow into the lesions and enhance the power of thermal ablation, thus improving the treatment efficacy of the following MWA (25).

Moreover, our study has some instructive information about HCC patients receiving cTACE combined with MWA as a bridging therapy to liver transplantation. Liver transplantation is by far the most effective therapy for liver cancer, and patient selection has resulted in remarkable 10-year post-liver transplantation survival rates for HCC patients within the Milan criteria. However, many HCC patients who were suitable for transplantation according to the Milan criteria dropped out while waiting for the transplanted liver source, causing disease progression. To receive increased allocation priority, HCC patients who are listed for liver transplantation are often treated while on the waiting list with loco-regional therapy (LRT) such as ablation and/or TACE (26). Moreover, patients who showed a complete response (CR) to LRT in the first follow-up imaging study were more likely to undergo liver transplantation. All the patients enrolled in our study were within Milan criteria, and all the patients achieved technical success and CR on the first follow-up enhanced MR. Our results demonstrated no significant difference in local tumour progression between the GA and LA groups. This result indicated that both local anaesthesia plus intraoperative sedation and general anaesthesia are effective anaesthesia modalities for CT-guided ablation in suitable HCC patients according to the Milan criteria. However, the liver transplantation rate after combined therapy failed to record causing a short follow-up period and considerable censored data. From the current results of our study, we deduced that the anaesthesia modality in the MWA procedure will not affect the success rate or therapeutic efficacy of subsequent liver transplantation. However, further research is warranted to confirm this hypothesis.

In the stratification analysis of LRFS of lesions, no significant differences were observed between different anaesthesia modalities. HCC lesions in perivascular and/or subcapsular locations were deemed as unfavourable locations. Heat sink effects were common for perivascular lesions, and large vessels with higher flow could draw away heat from the ablative area. Theoretically, perivascular lesions might benefit more from general anaesthesia than local anaesthesia during the ablation procedure. The possible reason for this discrepancy may be that all the HCC lesions enrolled in our study were treated by MWA, and the MWA procedure could create high-temperature heating and lack of heat sink effects (27, 28). Moreover, lipiodol deposited in the HCC lesions, as aforementioned, could diminish hepatic artery inflow and reduce the “heat-sink” effect, thus enhancing the efficacy of subsequent ablation (25, 29).

Subcapsular location is challenging for percutaneous ablation due to the difficulty of accurately inserting the ablation needle and obtaining sufficient ablative margin along the hepatic capsule. On the other hand, an underlying thermal injury of adjacent structures for subcapsular lesions is associated with a higher risk of major complications. The lack of a significant difference in LRFS between the GA and LA groups for subcapsular lesions might be attributed to the following reasons. In our study, artificial ascites was used, regardless of the anaesthesia modality, to allow hydrodissection for subcapsular lesions as appropriate (30). Consequently, the incidence of treatment-related complications was reduced while the ablation efficacy improved. On the other hand, deposited lipiodol in HCC lesions enhanced the visibility of subcapsular lesions thus improving the accuracy of ablation antenna insertion (25).

There was no significant difference in the occurrence of treatment-related AEs and complications before and after PSM. This result demonstrated that both general and local anaesthesia was safe and feasible anaesthesia modalities during the MWA procedure. Regarding post ablation anaesthesia AEs, the GA group showed obviously higher incidences of dizziness, urinary retention and respiratory depression than the LA group, but no serious anaesthesia AEs (grades≥3) occurred in either groups. The cost analysis showed that the GA group had a longer MWA procedure time, more participating healthcare providers, and more hospitalization costs. Notably, both the GA and LA groups had comparable hospital stays, which may be attributed to the lack of a significant difference in the occurrence of treatment-related AEs and complications. In this regard, general anaesthesia is more costly than local anaesthesia but shows comparable treatment efficacy and safety.

There are several limitations in the present study. First, although propensity score-matching (PSM) was applied to diminish potential confounding and selection bias, due to the retrospective and single-center nature of our study, there was still some heterogeneity between the two anaesthesia groups. Second, the details of the MWA procedure, such as the power and ablation time, were not recorded specifically. This drawback might hamper the rigor of the results in our study, as the parameters of the MWA procedure were critical for the therapeutic efficacy. Third, TACE performed before MWA may increase the rate of AEs and complications related to the MWA procedure. Finally, the limited follow-up period and population of our study may have impeded the thorough survival assessment of the patients. The long-term therapeutic outcomes of the two anaesthesia groups during the MWA procedure need further investigation.

Overall, our retrospective study demonstrated comparable therapeutic outcomes between general anaesthesia and local anaesthesia plus sedation for the MWA procedure. Moreover, both anaesthesia modalities during the MWA procedure were safe and effective for HCC patients within the Milan criteria. However, the costs of both procedure time, participating healthcare providers, hospitalization costs and anaesthesia AEs of general anaesthesia were higher than those of local anaesthesia. Thus, local anaesthesia plus sedation may be more adaptive to CT-guided MWA for HCC patients within Milan criteria who receive combination therapy for curative purposes.

## Data availability statement

The original contributions presented in the study are included in the article/**Supplementary Material**. Further inquiries can be directed to the corresponding author.

## Ethics statement

The studies involving humans were approved by The Second Xiangya Hospital of Central South University. The studies were conducted in accordance with the local legislation and institutional requirements. The ethics committee/institutional review board waived the requirement of written informed consent for participation from the participants or the participants’ legal guardians/next of kin because Due to the retrospective nature of the present study.

## Author contributions

J-YZ, DZ, Z-LT and H-QL analyze and interpret the patients’ data and review the patients’ images. J-YZ and Z-LT collect the data. H-QL, DZ and J-YZ revise the manuscript. J-YZ and H-QL are major contributors to writing the manuscript. H-QL provides the concept and is a major contributor to the manuscript editing. All authors contributed to the article and approved the submitted version.

## Supplementary material

Supplementary file 1 Baseline patients’ characteristics before propensity score matching (PSM).

Supplementary file 2 Baseline patients’ characteristics after propensity score matching (PSM).

Supplementary file 3 Local recurrence-free survival (LRFS) for lesions before propensity score matching (PSM).

Supplementary file 4 Local recurrence-free survival (LRFS) for lesions after propensity score matching (PSM).
